# Probable Psittacosis Outbreak Linked to Wild Birds

**DOI:** 10.3201/eid1103.040601

**Published:** 2005-03

**Authors:** Barbara L. Telfer, Sarah A. Moberley, Krishna P. Hort, James M. Branley, Dominic E. Dwyer, David J. Muscatello, Patricia K. Correll, John England, Jeremy M. McAnulty

**Affiliations:** * New South Wales (NSW) State Department of Health, NSW, Australia; † Wentworth Public Health Unit, NSW, Australia; ‡ Nepean and Blue Mountains Pathology Service, NSW, Australia; § Westmead Hospital, NSW, Australia; ¶ Blue Mountains District ANZAC Memorial Hospital, NSW, Australia

**Keywords:** Chlamydophila psittaci, community acquired pneumonia, outbreak, Psittacosis, research

## Abstract

In autumn 2002, an outbreak of probable psittacosis occurred among residents of the Blue Mountains district, Australia. We conducted a case-control study to determine independent risk factors for psittacosis by comparing exposures between hospitalized patients and other residents selected randomly from the telephone directory. Of the 59 case-patients with laboratory results supportive of psittacosis, 48 participated in a case-control study with 310 controls. Independent risk factors were residence in the upper Blue Mountains (odds ratio [OR] = 15.2, 95% confidence interval [CI] 5.6–41.7), age of 50–64 years (OR = 3.9, 95% CI 1.5–10.5), direct contact with wild birds (OR = 7.4, 95% CI 2.5–22), and mowing lawns without a grass catcher (OR = 3.2, 95% CI 1.3–8.0). Protective equipment is recommended for residents in areas frequented by free-ranging birds if contact with birds and their droppings is likely when performing outdoor activities such as lawn mowing.

## Abstract 2

Residence in the upper Blue Mountains, age of 50–64 years, direct contact with wild birds, and lawn mowing without a grass catcher were associated with psittacosis.

Psittacosis is a human disease caused by infection with the bacterium *Chlamydophila psittaci*. The bacterium also causes avian chlamydiosis, a disease reported in psittacine birds such as parrots, cockatiels, and parakeets (*1*–*3*). *Chlamydophila psittaci* can be present in large numbers in the droppings of sick birds and in dust contaminated by infected droppings (*4*). The organism can remain infectious in the environment for months (*1*). Human infection usually occurs when a person inhales the bacterium shed in feces and secretions of infected birds (*1*–*3*,*5*). Sheep, goats, cattle, and reptiles can also be infected, but these animals have rarely been linked to human cases (*1*,*3*,*6*).

Psittacosis has an incubation period of 1 to 4 weeks, and manifestations of disease can range from asymptomatic infection to systemic illness with severe pneumonia (*1*,*5*,*7*). Untreated psittacosis has a reported case-fatality rate of 15% to 20% (*1*,*3*). Psittacosis is most commonly reported among people in close contact with domestic birds, such as bird owners, poultry farmers, veterinarians, and workers within pet shops and poultry-processing plants (*1*–*3*,*5*,*8*–*13*). Sporadic cases and an outbreak in Australia linked to contact with free-ranging (wild) birds have been reported; however, little information is available on the role of wild birds in the transmission of *Chlamydophila psittaci* to humans (*14*,*15*)

Psittacosis became a notifiable disease in New South Wales (NSW), Australia, in 2001, and 38 laboratory notifications were received by the state health department that year, an incidence of 5.7 cases per 1,000,000 population for NSW (*16*,*17*). In May 2002, clinicians at the Blue Mountains Hospital (BMH), in the Wentworth Area Health Service, NSW, a 1-hour drive west of Sydney’s central business district, reported an increase in adult admissions for severe community-acquired pneumonia. In March to May 2002, a total of 160 persons with pneumonia were seen at the BMH emergency department, compared with 82 in March to May 2001. The population of the Blue Mountains is ≈80,000 persons, and the area includes a large national park. The lower Blue Mountains (altitude ≈160 m) is on the western outskirts of Sydney, and residences tend to have suburban-style yards. The upper Blue Mountains district (altitude ≈1,044 m) lies further west, receives more rain, and has more bush land; its residential areas have larger yards and are closer to bush land. Reports that patients had found increased numbers of dead free-ranging birds in their yards, handled dead birds, and occasionally mowed over dead bird carcasses prompted clinicians to suspect psittacosis, although no case had been confirmed by laboratory testing. We report on our investigation into the extent and most likely cause of this outbreak.

## Methods

We defined a suspected case of disease as community-acquired pneumonia (as primary clinical diagnosis), confirmed by chest radiograph, in a resident of the Blue Mountains 15 to 75 years of age who was admitted to a local hospital between March 1 and June 30, 2002. Patients with a history of congestive cardiac failure and chronic obstructive pulmonary disease were excluded. Active surveillance for suspected cases of psittacosis was initiated in the first week of June 2002. Surveillance was performed by review of patient medical records and daily contact with the emergency department and infection control staff at the Blue Mountains and other local hospitals. We attempted to contact all suspected case-patients by telephone to invite them to participate in the study and provide serum samples for laboratory testing.

We defined a probable case of disease as psittacosis in a suspected case-patient in which any of the following were demonstrated: seroconversion, a 4-fold rise in immunoglobulin (Ig) G titer by microimmunofluorescence (MIF), or a single or static high convalescent-phase MIF IgG titer to *Chlamydophila psittaci*.

We conducted a case-control study to identify independent risk factors for psittacosis in the outbreak. Only probable case-patients were included in the analysis. To identify controls, random digit dialing was used to select household telephone numbers from the randomly sorted Blue Mountains telephone directory. All randomly selected households were telephoned, and 1 person 15–75 years of age from every household was randomly selected and invited to participate in the study (*18*,*19*).

Interviews were conducted by trained interviewers in a computer-assisted telephone interview service, from June 18 to July 2, 2002, 7 days per week, during the day and the evening. Up to 10 attempts were made to contact each case-patient and control. Case-patients and controls completed a detailed telephone questionnaire, which included questions on demographics; contact with poultry, pet, and free-ranging birds; types of bird contact; other animal contact; and gardening and other outdoor activities undertaken in the 3 weeks before onset of illness (for case-patients) or the 3-week period April 1–21, 2002 (for controls).

Statistical analysis was conducted by using SAS Version 8.02 (SAS Institute Inc., Cary, NC, USA) (*20*). In univariate analysis, we compared characteristics and potential risk factors reported by probable case-patients and controls. Univariate analysis was performed by using chi-square tests and logistic regression analysis. When expected cell counts were <5, Fisher exact 2-sided p value was used. We performed multivariable logistic regression modeling using the backward stepwise elimination method to identify independent risk factors for psittacosis (*21*).

Serum samples were tested for *Chlamydia* and *Chlamydophila* species IgG (to endpoint titer) with a MIF assay (Chlamydia IgG SeroFIA, Savyon Diagnostics, Ashdod, Israel) using purified *Chlamydophila pneumoniae*, *Chlamydia trachomatis*, and *Chlamydophila psittaci* elementary bodies as antigen and a complement fixation (CF) assay using a *Chlamydia* genus-specific glycoprotein (Virion, Ruschlikon, Switzerland) (*22*,*23*). According to the manufacturer’s instructions, MIF titers >1:64 for *Chlamydophila psittaci* and *Chlamydia trachomatis* and >1:512 for *Chlamydophila pneumoniae* were regarded as indicative of current or recent infection. Serum samples were also tested for *Chlamydia trachomatis* IgG and IgA antibodies by using an enzyme immunoassay (EIA) (Medac Diagnostika, Wedel, Germany). All paired samples were tested in parallel in a single laboratory. Complement-fixing antibodies to *Mycoplasma pneumoniae*, influenza A and B viruses, adenovirus, and *Coxiella burnetii* were determined. *Legionella pneumophila* serogroups 1–6 and *L. longbeachae* total antibodies were tested by using an in-house immunofluorescence assay. In the later stage of the outbreak, upper respiratory tract specimens were collected from patients with pneumonia for *Chlamydophila pneumoniae* polymerase chain reaction (PCR) and for respiratory virus isolation. PCR for *Chlamydophila pneumoniae* was performed according to the published method (*24*).

To identify birds with avian chlamydiosis, we invited all local veterinarians and wildlife workers by letter to submit sick or dead birds for testing at a regional veterinary laboratory. The avian diagnostic test was based on direct immunofluorescence of a conjugated monoclonal antibody against a common, shared, genus-specific lipopolysaccharide antigen (Chlamydia-CEL VET IF TEST, Cellabs Diagnostics Pty Ltd, Brookvale, NSW, Australia). Household contacts of all probable case-patients were asked to submit any birds that the patient was known to have had contact with before onset of illness.

## Results

Of 95 suspected cases identified, 59 (62%) had serologic evidence of psittacosis (probable cases), 30 (32%) were seronegative, and 6 (6%) were lost to follow up. The first patient with a probable case of psittacosis was hospitalized on March 11, 2002, marking the start of the outbreak, which peaked in late April to early May, with the last probable case-patient admitted to the hospital on June 29 (Figure). Of the 59 patients with probable cases of psittacosis, 36 (61%) were men, 50 (85%) resided in the upper Blue Mountains (altitude 770–1044 m), and 32 (54%) were 50–64 years of age. The average length of hospital stay was 7 days (range 2–29). No deaths occurred, although 2 patients required intensive care with intubation and mechanical ventilation.

**Figure:**
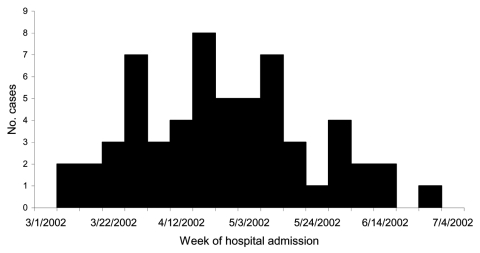
Hospitalized patients with cases of probable psittacosis, Blue Mountains, by week of hospital admission.

### Case-Control Study

Of the 59 probable case-patients, 48 (81%) were interviewed for the case-control study, 2 refused to participate, 2 could not be contacted, and 7 were on vacation. Of 384 eligible controls contacted, 310 (80%) agreed to participate.

Symptoms reported by the 48 case-patients included fever (25 patients), weakness and tiredness (24 patients), sweats and chills (20 patients), aching muscles (16 patients), headache (16 patients), shortness of breath (9 patients), dry cough (8 patients), confusion (5 patients), vomiting (6 patients), diarrhea (5 patients), sore throat (3 patients), and loose cough (2 patients). In univariate analysis, when compared with controls, case-patients were significantly more likely to be male, to be 50–64 years of age, and to reside in the upper Blue Mountains (Table 1). Case-patients were also significantly more likely than controls to report direct contact with live or dead free-ranging birds, and a dose-response relationship existed between reported level of contact with free-ranging birds and disease (chi-square test for linear trend = 24.5, p < 0.001). In addition, case-patients were significantly more likely than controls to report lawn mowing without a grass catcher and to have spent more time performing this activity. Conversely, case-patients were significantly less likely than controls to report contact with caged or domestic birds, a history of asthma, and potting or weeding in the yard.

**Table 1:** Potential risk factors for psittacosis among case-patients and controls in the Blue Mountains outbreak, Australia

Potential risk factor	Case-patients, N = 48 (%)	Controls, N = 310 (%)	Crude OR (95% CI)*	p value
Age group (y)				
15–49	10 (21)	155 (50)	Referent	
50–64	27 (56)	89 (29)	4.7 (2.2–10.2)	0.0007
65–75	11 (23)	66 (21)	2.6 (1.0–6.4)	0.04
Male sex	30 (63)	125 (40)	2.5 (1.3–4.6)	0.005
Resident of the upper Blue Mountains	42 (88)	104 (34)	13.9 (5.7–33.7)	<0.0001
History of asthma	1 (2)	64 (21)	0.08 (0.01–0.6)	0.0009
Employed	28 (58)	192 (62)	0.9 (0.5–1.6)	0.6
Outdoor employment	7 (15)	39 (13)	1.2 (0.5–2.8)	0.64
Bird contact				
Contact with caged or domestic birds	3 (6)	56 (18)	0.3 (0.1–1.0)	0.04
Contact with poultry birds	3 (6)	28 (9)	0.7 (0.2–2.3)	0.80
Visit to pet shop	9 (20)	39 (13)	1.6 (0.7–3.6)	0.25
Visit to aviary	0 (0)	3 (1)	Incalculable	1.0
Visit to zoo	0 (0)	3 (1)	Incalculable	1.0
Visit to poultry farm	0 (0)	4 (1)	Incalculable	1.0
Level of contact with free-ranging (wild) birds†				
No contact	9 (19)	137 (44)	Referent	
Only indirect contact‡	15 (31)	121 (39)	1.9 (0.8–4.5)	0.15
Any direct contact§	24 (50)	52 (17)	7.0 (3.1–16.1)	<0.0001
Yard exposures				
Lawn mowing				
Did not mow lawn	21 (44)	170 (55)	Referent	
Only mowed lawn with a grass catcher	4 (8)	75 (24)	0.4 (0.1–1.3)	0.14
Mowed lawn without a grass catcher	23 (48)	65 (21)	2.9 (1.5–5.5)	0.0017
Mowed lawn without a catcher for >1 hour	15 (31)	20 (6)	6.6 (3.1–14.1)	<0.0001
Pruning, cutting back branches	21 (45)	141 (47)	1.0 (0.5–1.7)	0.88
Potting or weeding	15 (31)	155 (50)	0.5 (0.2–0.9)	0.02
Watering yard	24 (50)	157 (52)	0.9 (0.5–1.7)	0.88
Raking yard	21 (45)	106 (35)	1.5 (0.8–2.8)	0.25
Soft landscaping	12 (25)	97 (31)	0.7 (0.4–1.5)	0.40
Hard landscaping	5 (10)	17 (5)	2.0 (0.7–5.7)	0.19
Mulching	9 (20)	56 (18)	1.1 (0.5–2.4)	0.84
Using compost	13 (27)	49 (16)	2.0 (1.0–4.0)	0.07
Using fertilizer	9 (20)	52 (17)	1.2 (0.5–2.6)	0.68
Home renovations or demolition	4 (9)	29 (9)	0.9 (0.3–2.7)	1.00
Bushwalking	13 (28)	91 (30)	1.0 (0.5–1.9)	1.00
Current or ex-smoker	23 (48)	141 (45)	1.1 (0.6–2.0)	0.76
General health self-rating				
Excellent to very good	23 (48)	182 (59)	Referent	
Good to fair	18 (37)	111 (36)	1.3 (0.7–2.5)	0.46
Poor to very poor	7 (15)	17 (5)	3.3 (1.2–8.7)	0.02
Yard attached to residence	48 (100)	302 (97)	Incalculable	0.61
Lived adjacent to bushland	33 (68)	184 (59)	1.5 (0.8–2.9)	0.27
Lived in a house	48 (100)	305 (98)	Incalculable	1.00
Lived in the Blue Mountains <5 years	11 (23)	38 (12)	2.1 (1.0–4.5)	0.07

*OR, odds ratio; CI, confidence interval. †Chi-square test for linear trend = 24.5, p value < 0.001. ‡Indirect contact only with free-ranging (wild) birds: watching bird at bird feeder, watching birds in yard, seeing dead birds, watching birds in bird bath, bird flying in house. §Any direct contact with free-ranging (wild) birds: catching birds, clipping birds’ feathers, tending to sick birds, touching birds’ feathers, cleaning up or touching birds’ nest, cleaning up or touching bird droppings, handling dead birds.

Case-patients were more likely than controls to report direct or indirect contact with the following bird species of the parrot family: crimson rosellas (odds ratio [OR] = 4.4, 95% confidence interval [CI] 2.3–8.7, p < 0.0001), king parrots (OR = 3.6, 95% CI 1.9–6.7, p < 0.0001), and gang gangs (OR = 2.6, 95% CI 1.1–6.0, p = 0.03). Case-patients were also more likely than controls to report direct or indirect contact with doves (OR = 4.2, 95% CI 1.8–10.3, p = 0.003), currawongs (OR = 4.0, 95% CI 2.1–7.4, p < 0.0001), and magpies (OR = 2.4, 95% CI 1.3–4.5, p = 0.004). Case-patients most commonly reported contact with crimson rosellas (73% of cases) and king parrots (60% of cases). Bird species were not included in multivariate analysis due to the likely unreliability of bird species identification.

In the multivariable logistic regression model, risk factors independently and positively associated with psittacosis were residence in the upper Blue Mountains, age of 50–64 years, direct contact with live or dead free-ranging birds, and lawn mowing without a grass catcher (Table 2). Risk factors independently and negatively associated with psittacosis were contact with caged or domestic birds, a history of asthma, and potting and weeding.

**Table 2:** Multivariable logistic regression model of potential risk factors for psittacosis among 48 case-patients and 310 controls in the Blue Mountains outbreak, Australia

Potential risk factors for psittacosis	Adjusted* OR (95% CI)†	p value
Age group (y)		
15–49	Referent	
50–64	3.9 (1.5–10.5)	0.006
65–75	2.8 (0.9–8.8)	0.08
Resident of the upper Blue Mountains	15.2 (5.6–41.7)	<0.0001
History of asthma	0.1 (0.01-0.8)	0.03
Level of contact with free-ranging (wild) birds		
No contact	Referent	
Only indirect contact	2.6 (1.0–7.3)	0.06
Any direct contact	7.4 (2.5–22)	0.0003
Contact with caged or domestic birds	0.2 (0.04–0.8)	0.02
Lawn mowing		
Did not mow lawn	Referent	
Only mowed lawn with a grass catcher	0.4 (0.1–1.3)	0.12
Mowed lawn without a grass catcher	3.2 (1.3–8.0)	0.01
Potting or weeding	0.2 (0.1–0.5)	0.001

*After adjustment for the effects of age group, sex, region of residence, outdoor employment, history of asthma, level of free-ranging (wild) bird contact, contact with caged or domestic birds, lawn mowing, potting or weeding, smoking history, general health self-rating, duration lived in the Blue Mountains, soft landscaping, hard landscaping, using compost, and residence adjacent to bushland. †OR, odds ratio; CI, confidence interval.

A number of persons with probable cases reported finding an unusually high number of dead free-ranging birds in their yards before onset of their illness; however, the bird carcasses had been disposed of before the investigation began. By the time the process for submission of birds for testing was arranged, winter had begun and few sick or dead birds were found. Of the 4 sick and 4 dead free-ranging birds submitted from the Blue Mountains area for testing, tissue of 1 king parrot tested positive for *Chlamydophila psittaci*.

### Laboratory Results

Of the 59 seropositive case-patients, 35 (59%) had a seroconversion or a 4-fold rise in *Chlamydophila psittaci* MIF IgG titer, and 24 (41%) without acute-phase serum samples had elevated *Chlamydophila psittaci* MIF IgG titers in 1 or more convalescent-phase samples. Of these 24, 9 (15%) had a static high titer in 2 convalescent samples, 2 (3%) had high (but different) titers in 2 samples, and 13 (22%) had a high titer in a single convalescent sample. No evidence of infection by other respiratory pathogens was obtained in any of the *Chlamydophila psittaci*–seropositive patients. Alternative microbiologic diagnoses were made in 8 of the 30 (27%) *Chlamydophila psittaci*–seronegative patients, including 4-fold rises in serum antibodies specific to *M. pneumoniae* (2 cases), *L. longbeachae* (1 case), *L. pneumophila* serogroup 1 (1 case), and influenza B (2 cases). In 2 cases, *Streptococcus pneumoniae* was cultured from blood or sputum. *Chlamydophila pneumoniae* PCR and respiratory virus isolation were performed on upper respiratory tract samples taken from 8 case-patients when they arrived at the hospital, and all results were negative; later in the investigation, all 8 had serologic evidence of *Chlamydophila psittaci* infection.

Of the 48 probable case-patients included in the case-control study, 28 (58%) had a seroconversion or a 4-fold rise in *Chlamydophila psittaci* MIF IgG titer, 8 (17%) had a static high titer in 2 samples, 2 (4%) had high (but different) titers in 2 convalescent-phase samples, and 10 (21%) had a high titer in a single convalescent-phase sample.

Of the 35 patients with probable cases who seroconverted or showed a 4-fold rise in *Chlamydophila psittaci* MIF IgG titer, 33 (94%) also had 4-fold rises in CF antibodies with a *Chlamydia* genus–specific glycoprotein, 1 had no rise, and 1 had insufficient serum for testing. In contrast, 3 of the 30 (10%) probable case-patients who were seronegative to *Chlamydophila psittaci* by MIF had 4-fold rises in CF antibodies.

Of the 59 probable case-patients with elevated *Chlamydophila psittaci* MIF antibody titers, 57 also had detectable *Chlamydia trachomatis* MIF IgG, generally at a lower titer. However, when an EIA was used, only 2 had *Chlamydia trachomatis* IgA, and 7 (12%) had *Chlamydia trachomatis* IgG detected. A similar proportion (10%) of the suspected case-patients who were seronegative for *Chlamydophila psittaci* had *Chlamydia trachomatis* IgG detected. Among the 59 probable case-patients, 21 had elevated *Chlamydophila pneumoniae*–specific IgG titers on MIF testing, although 7 showed a 4-fold rise or higher, and all were less than the rises in *Chlamydophila psittaci*.

Active surveillance identified 2 persons with laboratory evidence of psittacosis who were linked to the Blue Mountains outbreak but resided elsewhere. One person had stayed in a vacation home there for most weekends in the months preceding the outbreak. The other lived elsewhere in Australia but had been on vacation in the Blue Mountains in the weeks preceding the outbreak. Between March and June 2003, 11 other persons with psittacosis were identified in NSW; these patients all reported other exposures, such as contact with pet birds and aviaries.

### Intervention

Media releases on June 11 and 12, 2002, identified psittacosis as a possible cause of the outbreak and advised residents of the Blue Mountains to avoid contact with free-ranging birds and their droppings, feathers, carcasses, and dust and to wear a particulate face mask and follow other prevention measures when performing outdoor activities likely to bring them in contact with free-ranging birds (*1*). Information on psittacosis and the outbreak were communicated by a telephone hotline, health department Web sites, and by fax to all medical doctors and hospitals in the Blue Mountains and adjacent regions (*25*)

## Discussion

We identified a large outbreak of probable psittacosis among residents of a forested district of Australia. Contact with live or dead free-ranging birds and lawn mowing without a grass catcher explained 70% of the cases. Possible reasons for the cessation of the outbreak include the onset of colder winter weather, which may reduce the prevalence or transmissibility of the infection among free-ranging birds and yard exposure among humans.

This study had several limitations. Case-patients and controls were exposed to media speculation over the role of parrots in the outbreak, so that people who believed they had psittacosis may have been more likely to report parrot exposure. Among patients, 28 completed hypothesis-generating questionnaires early in the investigation (between May 30 and June 7), which may have provided them with additional prompts to recall parrot exposure. However, diagnosis was unknown at the time patients completed the questionnaire, and few had laboratory evidence of psittacosis at the time they completed the questionnaire. Potential recall bias may also be countered because similar proportions of case-patients and controls reported no difficulty recalling contact with birds (case patients = 83%, controls = 85%, OR = 0.9, 95% CI 0.4–2.0, p = 0.7).

The serologic diagnosis of chlamydial infections is difficult because many assays lack specificity, and published data on the sensitivity and specificity of the MIF assay used in this study are limited (*26*). In this study, acute *Chlamydophila psittaci* infection, rather than *Chlamydophila pneumoniae* or *Chlamydia trachomatis* infection, was supported by 3 factors: 1) the seroconversion and 4-fold rises in *Chlamydophila psittaci* MIF IgG titers in samples from most case-patients (with most also having 4-fold rises in CF antibodies to *Chlamydia*), compared with lower MIF IgG titers to *Chlamydophila pneumoniae* and *Chlamydia trachomatis*, 2) the similar background rates of *Chlamydia trachomatis*–specific IgG on EIA in samples from the *Chlamydophila psittaci* seropositive probable and seronegative suspected case-patients, and 3) history of bird exposure among case-patients.

Analysis of hospital admission and discharge data indicated that, compared with the NSW state average and with the lower Blue Mountains average, increased rates of pneumonia have been seen among residents of the upper Blue Mountains during the autumn of previous years (unpub. data). This excess in pneumonia may be due to seasonal outbreaks of psittacosis in the area. Free-ranging birds are plentiful in the Blue Mountains, and chlamydiosis has been clinically diagnosed among juvenile birds, in particular crimson rosellas, in the area during the autumn of previous years (M. Cannon, pers. comm.).

Reasons for the positive association between probable psittacosis and residence in the upper Blue Mountains are speculative. The residents of the upper Blue Mountains are slightly older than that of the lower Blue Mountains (unpub. data) and may be more likely to remain at home and be active in their yards than the slightly younger residents of the lower Blue Mountains, who tend to commute to work in Sydney every day. This age difference may result in greater proximity between residents and free-ranging birds in the upper Blue Mountains.

Although the reason probable psittacosis case-patients were more likely to be 50–64 years of age is unclear, younger people may have had milder infection or been less likely to seek medical attention and require hospitalization (*27*,*28*). That persons with congestive cardiac failure or chronic obstructive pulmonary disease were excluded from the study may explain why persons 65–75 years of age appeared less at risk of disease.

Why a history of asthma seemed protective against psittacosis is uncertain. Persons with a history of asthma may be less likely to perform outdoor yard activities that generate dust and pollen. That persons who reported contact with caged or domestic birds appeared protected against disease may be related to previous exposure to *Chlamydophila psittaci*. While the development of protective immunity following infection with *Chlamydophila psittaci* has not been proven, laboratory-confirmed cases of reinfection have seldom been reported (*2*,*29*). That disease was less likely to develop in persons who reported potting or weeding may be because these activities generate little contaminated dust compared with activities such as lawn mowing.

Reported symptoms of the probable psittacosis case-patients in this outbreak were similar to those described elsewhere (*1*–*3*,*5*,*10*,*15*). One other community-wide outbreak of psittacosis has been reported to be potentially associated with contact with free-ranging birds (*15*). In 1995, 16 cases of psittacosis among residents of a rural town in a forested area in southern Australia were linked to trimming and mowing lawns and time spent in a yard, which are thought to be proxies for exposure to infectious particles shed by free-ranging birds with chlamydiosis (*15*). While this 1995 study did not find a significant relationship between illness and handling of dead birds (OR 2.4, 95% CI 0.25–21.05), the OR supports the positive association between illness and handling live or dead free-ranging birds (adjusted OR 7.4, 95% CI 2.5–22, p < 0.001) found in our study. The 1995 study reported the risk of illness associated with lawn mowing was not linked to use of a grass catcher, though whether the authors differentiated between those who never used a catcher and those who sometimes used a catcher is not clear. Previous studies have linked lawn mowing with psittacosis and with primary pneumonic tularemia (*15*,*30*). In our case-control study questionnaire, we differentiated between persons who mowed only with, with and without, and only without a grass catcher. We found this probable psittacosis outbreak to be linked to lawn mowing only without the use of a grass catcher. We could find no evidence in the literature suggesting that the use of a grass catcher provides lawn mower operators some protection from inhaling infectious particles, though grass catcher marketing materials of lawn mower manufacturers in Australia suggest that grass catchers reduce the generation of dust and hence the amount of dust expelled into the operator’s breathing zone (*31*).

This outbreak could have been missed altogether if not for alert clinicians. Laboratory evidence of psittacosis requires acute-phase and convalescent-phase (collected at least 6 weeks after onset) species-specific serologic testing for *Chlamydophila* or *Chlamydophila psittaci* PCR or culture. These tests require request by the treating doctor and referral to a specialized laboratory and enough concern to convince the patient to return for phlebotomy after recovery. In most clinical settings, underdiagnosis is therefore likely.

Clinicians should consider a diagnosis of psittacosis in persons with respiratory disease who reside in or have visited areas frequented by free-ranging birds. Health departments should also be alert for such outbreaks to facilitate diagnosis. Residents of areas frequented by free-ranging birds should avoid direct contact with dead or sick birds and bird droppings, use a particulate mask and gloves if contact is unavoidable, and avoid mowing lawns without a grass catcher. Further study is needed to identify effective measures, such as lawn mower design features, for reducing human exposure to *Chlamydophila psittaci* and other zoonoses.
